# Supplementary material for: Integration of hunger and hormonal state gates infant-directed aggression
Source: Nature. 2025 Oct 22;648(8092):138–45. doi: 10.1038/s41586-025-09651-2 (PMC12675289; doi:10.1038/s41586-025-09651-2)
Supplement: Supplementary file 1 — Supplementary Tables 1–3, Supplementary Note and Supplementary References. [file 41586_2025_9651_MOESM1_ESM.pdf]

nature portfolio

https://doi.org/10.1038/s41586-025-09651-2

Supplementary information

Integration of hunger and hormonal state gates infant-directed aggression

In the format provided by the
authors and unedited

Supplementary Table 1: Brain areas and coordinates. Injection, viral injection; stimulation, implantation coordinates for optic fibres (fibre tip position); recording, implantation coordinates for GRIN lenses or photometry fibres (tip position). Coordinates are AP (anteroposterior) / ML (mediolateral) / DV (dorsoventral) and in mm. DV coordinates are measured from brain surface.

Acronym	Brain area	Injection	Stimulation	Recording
AAA	Anterior amygdaloid area	–	–	–
ADP	Anterodorsal preoptic nucleus	–	–	–
AHA	Anterior hypothalamic area	–	–	–
ASO	Anterolateral striatal organ	–	–	–
ARC	Arcuate nucleus of hypothalamus	–1.5 / ±0.25 / –5.90	–	–1.5 / 0.3 / –5.75
AVP	Anteroventral preoptic nucleus	–	–	–
AVPV	Anteroventral periventricular nucleus	–	–	–
BLAa/p/v	Basolateral amygdaloid nucleus, anterior/posterior/ventral part	–	–	–
BNST	Bed nucleus of the stria terminalis	–	–	–
CEAc/l/m	Central amygdaloid nucleus, capsular/lateral/medial part	–	–	–
COAa/pl/pm	Cortical amygdaloid nucleus, anterior/posterolateral/posteromedial part	–	–	–
DMH	Dorsomedial hypothalamic nucleus	–	–	–
LA	Lateral amygdaloid nucleus	–	–	–
LH	Lateral hypothalamic area	–	–	–
LM	Lateral mammillary nucleus	–	–	–
LPOA	Lateral preoptic area	–	0.0 / ±1.0 / –4.65	–
LSc/r/v	Lateral septal nucleus, central/ventral part	–	–	–
MeA	Medial amygdaloid nucleus	–	–	–
MEPO	Median preoptic nucleus	–	–	–
MPO(A)	Medial preoptic area	0.0 / ±0.5 / –5.05	0.0 / ±0.5 / –4.65	0.0 / 0.5 / –4.85
MPN	Medial preoptic nucleus	–	–	–
MS	Medial septal nucleus	–	–	–
PA	Paraventricular thalamic nucleus, anterior part	–	–	–
PAG	Periaqueductal gray	–	–	–
PBN	Parabrachial nucleus	–	–	–

PD	Posterodorsal preoptic nucleus	–	–	–
PeF	Perifornical nucleus	–	–	–
PH	Posterior hypothalamic area	–	–	–
PMv	Ventral premammillary nucleus	–	–	–
PS	Parastrial nucleus	–	–	–
PVa/i/p/po	Periventricular hypothalamic nucleus, anterior/intermediate/posterior/preoptic part	–	–	–
PVH	Paraventricular hypothalamic nucleus	–	0.7 / 0 / –4.4	–
PVT	Paraventricular nucleus of the thalamus	–	–	–
SCH	Suprachiasmatic nucleus	–	–	–
SUM	Supramammillary nucleus	–	–	–
TMd/v	Dorsal/ventral tuberomammillary nucleus	–	–	–
TU	Tuberal nucleus	–	–	–
VLPO	Ventrolateral preoptic nucleus	–	–	–
VMH	Ventromedial hypothalamic nucleus	–	–	–
VMPO	Ventromedial preoptic nucleus	–	–	–
VTA	Ventral tegmental area	–	–	–

Supplementary Table 2: Blood plasma levels of P4 and E2 for estrous and pregnancy stages. Maximal values for each state are shown. P, proestrus, O, oestrus, M, metestrus, D, diestrus, D10, pregnancy day 10, D18, pregnancy day 18.

State	P4 plasma level (ng/ml)	E2 plasma level (pg/ml)	Ref.	(P4/E2) × 10^3	Switching rate
P	64	10.3	[82, 83]	6.21	0.57
O	4	4.2	[82, 83]	0.95	0.31
M	27	3.1	[82, 83]	8.71	0.7
D	15	7.5	[82, 83]	2	0.46
D10	37	16	[84, 85]	2.31	0.44
D18	7	6.8	[84, 85]	1.03	0.33

Supplementary Table 3: Statistical analyses

Figure	Analysis type	Variables analysed	Test statistic / mean difference	P-value	DOF
Fig. 1b	Logistic Regression	Group (0, 120, 240, 360, 480)	69.44	1.11E-160	(4, 203)
Fig. 1b	Chi-square test	Group (0, 120, 240, 360, 480)	31.92	1.99E-06	4
Fig. 1b	Chi-square test	Group (480, Refed 1h, Refed 2h)	15.08	0.0005	2
Fig. 1c	U test	food intake Pre and Post	16.44	3.49E-30	NA
Fig. 1e	U test	Gq vs ctrl	49	0.002	NA
Fig. 1f	Fisher's exact test	Gq vs Ctrl	∞	0.005	NA
Fig. 1h	U test	Gly vs ctrl	3	0.015	NA
Fig. 1i	Fisher's exact test	Gly vs Ctrl	0.03	0.029	NA
Fig. 1k	Chi-square test	Group (acute, pre15', pre30', ctrl)	14	0.000912	2
Fig. 1k	Chi-square test	Group (pre 30' LPOA, PVH, ctrl)	4.36	0.113	2
Fig. 1k	Fisher's exact test with Benjamini-Hochberg	Group (acute, ctrl)	1.4	1	NA
Fig. 1k	Fisher's exact test with Benjamini-Hochberg	Group (pre15', ctrl)	1.17	1	NA
Fig. 1k	Fisher's exact test with Benjamini-Hochberg	Group (pre30', ctrl)	∞	0.00649	NA
Fig. 2b	One-way ANOVA	Group (ctrl, Agg+, Agg-)	117.51	6.25E-18	(2, 42)
Fig. 2b	Tukey post hoc test	Group (Agg+, Agg-)	-0.12	0.172	42
Fig. 2b	Tukey post hoc test	Group (Agg+, ctrl)	-0.65	<1e-50	42
Fig. 2b	Tukey post hoc test	Group (Agg-, ctrl)	-0.53	3.16E-12	42
Fig. 2c	One-way ANOVA	Group (ctrl, Agg+, Agg-)	16.47	0.0005	(2, 11)
Fig. 2c	Tukey post hoc test	Group (Agg+, Agg-)	1.34	0.391	11
Fig. 2c	Tukey post hoc test	Group (Agg+, ctrl)	-3.96	0.0034	11
Fig. 2c	Tukey post hoc test	Group (Agg-, ctrl)	-5.3	0.0006	11
Fig. 2e	Chi-square test	Group (P-D)	9.02	0.029	3
Fig. 2e	Fisher's exact test with Benjamini-Hochberg	Group (P-E)	2.83	0.142	NA
Fig. 2e	Fisher's exact test with Benjamini-Hochberg	Group (P-M)	0.56	0.316	NA
Fig. 2e	Fisher's exact test with Benjamini-Hochberg	Group (P-D)	1.54	0.464	NA
Fig. 2e	Fisher's exact test with Benjamini-Hochberg	Group (E-M)	0.2	0.01	NA
Fig. 2e	Fisher's exact test with Benjamini-Hochberg	Group (E-D)	0.54	0.395	NA
Fig. 2e	Fisher's exact test with Benjamini-Hochberg	Group (M-D)	2.75	0.039	NA
Fig. 2f	F-Test for Overall Model Fit	P/E ratio	3.09	0.03	(4, 122)
Fig. 2h	Binomial test	PD10	0.44	1	NA
Fig. 2h	Binomial test	PD18	0.33	1	NA
Fig. 2j	Poisson binomial test	MPOA Esr1 KO	–	0.00039	NA
Fig. 2j	Poisson binomial test	MPOA PR KO	–	0.00351	NA
Fig. 2j	Poisson binomial test	MPOA ctrl	–	0.251	NA
Fig. 2j	Poisson binomial test	Arc Esr1 KO	–	0.285	NA
Fig. 2j	Poisson binomial test	Arc PR KO	–	0.391	NA
Fig. 3b	U test with Benjamini-Hochberg	Group (Pre, Agg+)	1635.5	0.0461	NA

Fig. 3b	U test with Benjamini-Hochberg	Group (Pre, Agg-)	1087	0.105	NA
Fig. 3b	U test with Benjamini-Hochberg	Group (Agg+, Agg-)	352.5	0.00222	NA
Fig. 3c	Fisher's exact test with Benjamini-Hochberg	Group (Agg+, Agg-)	3.68	0.175	NA
Fig. 3c	Fisher's exact test with Benjamini-Hochberg	Group (Pre, Agg+)	2.21	0.175	NA
Fig. 3c	Fisher's exact test with Benjamini-Hochberg	Group (Pre, Agg-)	0.6	0.442	NA
Fig. 3c	Fisher's exact test with Benjamini-Hochberg	Group (Pre, ZD)	0.05	0.0011	NA
Fig. 3c	Fisher's exact test with Benjamini-Hochberg	Group (Pre, NPY)	0.21	0.0255	NA
Fig. 3f	One-way ANOVA	Group (Pre, Agg+, Agg-)	3.47	0.034	(2, 141)
Fig. 3f	Tukey post hoc test	Group (Agg+, Agg-)	4.27	0.0563	141
Fig. 3f	Tukey post hoc test	Group (Agg+, Pre)	3.64	0.0376	141
Fig. 3f	Tukey post hoc test	Group (Agg-, Pre)	-0.63	0.893	141
Fig. 3f	U test with Benjamini-Hochberg	Group (Pre, ZD)	801	0.000239	NA
Fig. 3f	U test with Benjamini-Hochberg	Group (Pre, NPY)	806	0.0201	NA
Fig. 3i	Unpaired t-test	Group (KD-Y, ctrl)	4.59	0.006	5
Fig. 3k	U test	Group (Agg+, KD-Y)	103	0.023	NA
Fig. 3l	U test	Group (Agg+, KD-Y)	112.5	0.027	NA
Fig. 3m	Fisher's exact test	Group (Agg+, KD-Y)	2.29	0.32	NA
Fig. 3o	log-rank test	Group (KD-Y, ctrl)	3.78	0.0259	1
Fig. 3q	One-way ANOVA	Group (P, E, M, D)	4.31	0.00689	(3, 98)
Fig. 3q	Tukey post hoc test	Group (D, E)	-0.22	0.999	98
Fig. 3q	Tukey post hoc test	Group (D, M)	-5.51	0.0062	98
Fig. 3q	Tukey post hoc test	Group (D, P)	-1.66	0.733	98
Fig. 3q	Tukey post hoc test	Group (E, M)	-5.29	0.139	98
Fig. 3q	Tukey post hoc test	Group (E, P)	-1.43	0.934	98
Fig. 3q	Tukey post hoc test	Group (M, P)	3.86	0.0818	98
Fig. 3s	One-way ANOVA	Hcn1 Group (P, E, M, D)	6.09	0.0126	(3, 10)
Fig. 3s	Tukey post hoc test	Group (D, E)	85.24	0.0142	10
Fig. 3s	Tukey post hoc test	Group (D, M)	17.29	0.861	10
Fig. 3s	Tukey post hoc test	Group (D, P)	3.65	0.998	10
Fig. 3s	Tukey post hoc test	Group (E, M)	-67.95	0.0672	10
Fig. 3s	Tukey post hoc test	Group (E, P)	-81.59	0.0184	10
Fig. 3s	Tukey post hoc test	Group (M, P)	-13.64	0.925	10
Fig. 3s	One-way ANOVA	Hcn2 Group (P, E, M, D)	7.13	0.00761	(3, 10)
Fig. 3s	Tukey post hoc test	Group (D, E)	19.98	0.0054	10
Fig. 3s	Tukey post hoc test	Group (D, M)	3.91	0.817	10
Fig. 3s	Tukey post hoc test	Group (D, P)	7.27	0.345	10
Fig. 3s	Tukey post hoc test	Group (E, M)	-16.07	0.0305	10
Fig. 3s	Tukey post hoc test	Group (E, P)	-12.71	0.0698	10
Fig. 3s	Tukey post hoc test	Group (M, P)	3.36	0.873	10
Fig. 3v	Fisher's exact test	Group (ZD, ctrl)	0	0.00794	NA
Fig. 4c	One-way ANOVA	Group (Pre+, Agg+, Pre-, Agg-)	58.72	3.19E-34	(3, 778)
Fig. 4c	Tukey post hoc test	Group (Agg+, Pre+)	8.46E-18	<1e-50	778

Fig. 4c	Tukey post hoc test	Group (Agg-, Pre-)	-5.94E-18	<1e-50	778
Fig. 4d	Two-way ANOVA	Group (Agg+, Agg-)	15.26	0.000202	1
Fig. 4d	Two-way ANOVA	Time	1062.41	2.02E-46	1
Fig. 4d	Two-way ANOVA	Interaction	2.33	0.131	1
Fig. 4d	Two-way ANOVA	Residual	–	–	76
Fig. 4e	U test	Group (Agg+, Agg-), window = 2s	10122	0.00147	NA
Fig. 4k	One-way ANOVA	Group (S, G, A)	10.13	0.00265	(2, 12)
Fig. 4k	Tukey post hoc test	Group (A, S)	-0.55	0.0032	12
Fig. 4k	Tukey post hoc test	Group (A, G)	-0.46	0.0113	12
Fig. 4k	Tukey post hoc test	Group (S, G)	0.09	0.765	12
Fig. 4l	One-way ANOVA	Group (Neural, PC, shuffled)	249.6	1.67E-10	(2, 12)
Fig. 4l	Tukey post hoc test	Group (Neural, PC)	0.01	0.977	12
Fig. 4l	Tukey post hoc test	Group (Neural, shuffled)	-0.55	6.15E-10	12
Fig. 4l	Tukey post hoc test	Group (PC, shuffled)	-0.55	5.44E-10	12
Fig. 4m	U test	Sniff Agg+ vs Agg-	2	0.381	NA
Fig. 4m	U test	Groom Agg+ vs Agg-	4	1	NA
Fig. 4n	F-Test for Overall Model Fit	PC distance	261.78	0.0437	(2, 1)
EDF 1a	One-way ANOVA	Group (0, 120, 240, 360, 480)	42.01	6.51E-20	(4, 90)
EDF 1b	Chi-square test	Group (Par, Ign)	0.6	0.437	2
EDF 1c	U test	Group (Par, Ign)	621.5	0.902	NA
EDF 1d	U test	Group (Agg+, Agg-)	110	0.0207	NA
EDF 1e	U test	Sniff, Group (Agg+, Agg-)	37	0.138	NA
EDF 1e	U test	Groom, Group (Agg+, Agg-)	61	0.699	NA
EDF 1f	Fisher's exact test	Cricket Pre vs Post	1.97	0.685	NA
EDF 1f	Fisher's exact test	Male intruder Pre vs Post	1	1	NA
EDF 1f	Fisher's exact test	Female intruder Pre vs Post	1	1	NA
EDF 1g	Fisher's exact test	Pre vs Post	0	0.107	NA
EDF 1h	Two-way ANOVA	Group (Pre, Post)	2.7	0.113	1
EDF 1h	Two-way ANOVA	Group (Agg+, Agg-)	0.1	0.756	1
EDF 1h	Two-way ANOVA	Group (Agg+, Agg-: Pre, Post)	0.03	0.858	1
EDF 1h	Two-way ANOVA	Residual	–	–	24
EDF 1i	Two-way ANOVA	Group (Pre, Post)	0.12	0.731	1
EDF 1i	Two-way ANOVA	Group (Agg+, Agg-)	0.01	0.946	1
EDF 1i	Two-way ANOVA	Group (Agg+, Agg-: Pre, Post)	0	0.968	1
EDF 1i	Two-way ANOVA	Residual	–	–	30
EDF 1j	Chi-square test	Group (Food, Water, Light, Noval, All, All+Food)	30.47	1.19E-05	5
EDF 1j	Fisher's exact test with Benjamini-Hochberg	Group (Food, Water)	∞	0.00964	NA
EDF 1j	Fisher's exact test with Benjamini-Hochberg	Group (Food, Light)	∞	0.00633	NA
EDF 1j	Fisher's exact test with Benjamini-Hochberg	Group (Food, Novel)	∞	0.00766	NA
EDF 1j	Fisher's exact test with Benjamini-Hochberg	Group (Food, All)	1.3	1	NA
EDF 1j	Fisher's exact test with Benjamini-Hochberg	Group (Food, All+Food)	∞	0.00964	NA
EDF 1j	Fisher's exact test with Benjamini-Hochberg	Group (All, All+Food)	∞	0.0114	NA

EDF 2c	U test with Benjamini-Hochberg	ASO; Group (Agg+, Agg-)	5	0.242	NA
EDF 2c	U test with Benjamini-Hochberg	AAA; Group (Agg+, Agg-)	5	0.242	NA
EDF 2c	U test with Benjamini-Hochberg	AHA; Group (Agg+, Agg-)	5	0.242	NA
EDF 2c	U test with Benjamini-Hochberg	ADP; Group (Agg+, Agg-)	9	0.513	NA
EDF 2c	U test with Benjamini-Hochberg	AVPV; Group (Agg+, Agg-)	1	0.0765	NA
EDF 2c	U test with Benjamini-Hochberg	AVP; Group (Agg+, Agg-)	0	0.0459	NA
EDF 2c	U test with Benjamini-Hochberg	ARC; Group (Agg+, Agg-)	9	0.513	NA
EDF 2c	U test with Benjamini-Hochberg	BLAa; Group (Agg+, Agg-)	18	0.798	NA
EDF 2c	U test with Benjamini-Hochberg	BLAp; Group (Agg+, Agg-)	20	0.568	NA
EDF 2c	U test with Benjamini-Hochberg	BLAv; Group (Agg+, Agg-)	15	1	NA
EDF 2c	U test with Benjamini-Hochberg	BNST; Group (Agg+, Agg-)	8	0.451	NA
EDF 2c	U test with Benjamini-Hochberg	CEAc; Group (Agg+, Agg-)	15	1	NA
EDF 2c	U test with Benjamini-Hochberg	CEAl; Group (Agg+, Agg-)	15	1	NA
EDF 2c	U test with Benjamini-Hochberg	CEAm; Group (Agg+, Agg-)	8	0.451	NA
EDF 2c	U test with Benjamini-Hochberg	COAa; Group (Agg+, Agg-)	9	0.513	NA
EDF 2c	U test with Benjamini-Hochberg	COApl; Group (Agg+, Agg-)	15	1	NA
EDF 2c	U test with Benjamini-Hochberg	COApm; Group (Agg+, Agg-)	17	0.913	NA
EDF 2c	U test with Benjamini-Hochberg	PMd; Group (Agg+, Agg-)	5	0.242	NA
EDF 2c	U test with Benjamini-Hochberg	DMH; Group (Agg+, Agg-)	4	0.212	NA
EDF 2c	U test with Benjamini-Hochberg	LA; Group (Agg+, Agg-)	20	0.568	NA
EDF 2c	U test with Benjamini-Hochberg	LH; Group (Agg+, Agg-)	3	0.178	NA
EDF 2c	U test with Benjamini-Hochberg	LSc; Group (Agg+, Agg-)	18	0.798	NA
EDF 2c	U test with Benjamini-Hochberg	LSr; Group (Agg+, Agg-)	12	0.798	NA
EDF 2c	U test with Benjamini-Hochberg	LSv; Group (Agg+, Agg-)	9	0.513	NA
EDF 2c	U test with Benjamini-Hochberg	LM; Group (Agg+, Agg-)	5	0.376	NA
EDF 2c	U test with Benjamini-Hochberg	LPOA; Group (Agg+, Agg-)	0	0.0459	NA
EDF 2c	U test with Benjamini-Hochberg	MeA; Group (Agg+, Agg-)	10	0.568	NA
EDF 2c	U test with Benjamini-Hochberg	MPO; Group (Agg+, Agg-)	0	0.0459	NA
EDF 2c	U test with Benjamini-Hochberg	MPN; Group (Agg+, Agg-)	4	0.212	NA
EDF 2c	U test with Benjamini-Hochberg	MEPO; Group (Agg+, Agg-)	2	0.115	NA
EDF 2c	U test with Benjamini-Hochberg	MS; Group (Agg+, Agg-)	5	0.242	NA

EDF 2c	U test with Benjamini-Hochberg	PVN; Group (Agg+, Agg-)	7	0.376	NA
EDF 2c	U test with Benjamini-Hochberg	PVT; Group (Agg+, Agg-)	9	0.513	NA
EDF 2c	U test with Benjamini-Hochberg	PeF; Group (Agg+, Agg-)	4	0.212	NA
EDF 2c	U test with Benjamini-Hochberg	PAG; Group (Agg+, Agg-)	14	1	NA
EDF 2c	U test with Benjamini-Hochberg	PVa; Group (Agg+, Agg-)	7	0.376	NA
EDF 2c	U test with Benjamini-Hochberg	PVi; Group (Agg+, Agg-)	10	0.568	NA
EDF 2c	U test with Benjamini-Hochberg	PVp; Group (Agg+, Agg-)	7	0.376	NA
EDF 2c	U test with Benjamini-Hochberg	PVpo; Group (Agg+, Agg-)	4	0.212	NA
EDF 2c	U test with Benjamini-Hochberg	PH; Group (Agg+, Agg-)	10	0.568	NA
EDF 2c	U test with Benjamini-Hochberg	PA; Group (Agg+, Agg-)	14	1	NA
EDF 2c	U test with Benjamini-Hochberg	PD; Group (Agg+, Agg-)	12	0.798	NA
EDF 2c	U test with Benjamini-Hochberg	PS; Group (Agg+, Agg-)	0	0.0459	NA
EDF 2c	U test with Benjamini-Hochberg	SCH; Group (Agg+, Agg-)	7	0.376	NA
EDF 2c	U test with Benjamini-Hochberg	SUM; Group (Agg+, Agg-)	6	0.333	NA
EDF 2c	U test with Benjamini-Hochberg	TU; Group (Agg+, Agg-)	0	0.0459	NA
EDF 2c	U test with Benjamini-Hochberg	TMd; Group (Agg+, Agg-)	10	0.568	NA
EDF 2c	U test with Benjamini-Hochberg	TMv; Group (Agg+, Agg-)	17	0.913	NA
EDF 2c	U test with Benjamini-Hochberg	PMv; Group (Agg+, Agg-)	8	0.451	NA
EDF 2c	U test with Benjamini-Hochberg	VTA; Group (Agg+, Agg-)	14	0.949	NA
EDF 2c	U test with Benjamini-Hochberg	VLPO; Group (Agg+, Agg-)	8	0.451	NA
EDF 2c	U test with Benjamini-Hochberg	VMH; Group (Agg+, Agg-)	2	0.115	NA
EDF 2c	U test with Benjamini-Hochberg	VMPO; Group (Agg+, Agg-)	6	0.333	NA
EDF 2g	Linear regression	Food intake	10.86	0.0459	(1, 3)
EDF 2h	U test	Group (MPOA, PVH)	8	1	NA
EDF 2i	Repeated-measures ANOVA	Group (pre, stim, post)	9.55	0.008	(2,8)
EDF 2i	Pairwise t test (post hoc) with Benjamini-Hochberg	Group (pre, stim)	-4.51	0.0323	4
EDF 2i	Pairwise t test (post hoc) with Benjamini-Hochberg	Group (pre, post)	-0.68	0.536	4
EDF 2i	Pairwise t test (post hoc) with Benjamini-Hochberg	Group (stim, post)	3.48	0.038	4
EDF 2j	Repeated-measures ANOVA	Group (pre, stim, post)	8.28	0.008	2
EDF 2j	Pairwise t test (post hoc) with Benjamini-Hochberg	Group (pre, stim)	0.24	0.822	(2, 10)

EDF 2j	Pairwise t test (post hoc) with Benjamini-Hochberg	Group (pre, post)	-4.1	0.028	5
EDF 2j	Pairwise t test (post hoc) with Benjamini-Hochberg	Group (stim, post)	-2.93	0.0488	5
EDF 2k	Repeated-measures ANOVA	Group (pre, stim, post)	2.12	0.171	(2, 10)
EDF 2l	Repeated-measures ANOVA	Group (pre, stim, post)	11.31	0.005	(2, 8)
EDF 2l	Pairwise t test (post hoc) with Benjamini-Hochberg	Group (pre, stim)	-17.22	0.0002	4
EDF 2l	Pairwise t test (post hoc) with Benjamini-Hochberg	Group (pre, post)	0.93	0.406	4
EDF 2l	Pairwise t test (post hoc) with Benjamini-Hochberg	Group (stim, post)	3.46	0.0386	4
EDF 2m	One-way ANOVA	Group (Gq, Opto, FD)	1.35	0.268	(2, 48)
EDF 3c	Chi-square test	Group (P, E, M,D)	1.2	0.754	3
EDF 3d	One-way ANOVA	Group (P, E, M, D)	0.18	0.91	(3, 64)
EDF 3e	One-way ANOVA	Group (P, E, M, D)	0.87	0.467	(3, 34)
EDF 3f	One-way ANOVA	Group (P, E, M, D)	0.37	0.776	(3, 56)
EDF 3g	Linear regression	KO cells	11.09	0.0104	(1, 9)
EDF 3h	Linear regression	KO cells	0.89	0.377	(1, 8)
EDF 4c	Chi-square test	Group (Pre, Agg+, Agg-)	14.32	0.00634	4
EDF 4c	Fisher's exact test with Benjamini-Hochberg	Group (Pre, Agg+)	0.75	0.79	NA
EDF 4c	Fisher's exact test with Benjamini-Hochberg	Group (Pre, Agg-)	7.77	6.57E-10	NA
EDF 4c	Fisher's exact test with Benjamini-Hochberg	Group (Agg+, Agg-)	10.41	3.15E-12	NA
EDF 4d	One-way ANOVA	Group (Pre, Agg+, Agg-)	3.43	0.0354	(2, 141)
EDF 4d	Tukey post hoc test	Group (Agg+, Agg-)	-160.93	0.0669	141
EDF 4d	Tukey post hoc test	Group (Agg+, Pre)	-143.06	0.0354	141
EDF 4d	Tukey post hoc test	Group (Agg-, Pre)	17.87	0.941	141
EDF 4e	Linear regression	Sag (mV)	5.74	0.01	(1, 100)
EDF 4f	One-way ANOVA	Group (Pre, Agg+, Agg-)	0.37	0.692	(2, 164)
EDF 4g	One-way ANOVA	Group (Pre, Agg+, Agg-)	1.86	0.166	(2, 51)
EDF 4h	One-way ANOVA	Group (Pre, Agg+, Agg-)	0.75	0.475	(2, 109)
EDF 4i	One-way ANOVA	Group (Pre, Agg+, Agg-)	2.6	0.0795	(2, 100)
EDF 4j	One-way ANOVA	Group (Pre, Agg+, Agg-)	0.03	0.972	(2, 102)
EDF 4k	One-way ANOVA	Group (Pre, Agg+, Agg-)	0.72	0.489	(2, 71)
EDF 4l	One-way ANOVA	Group (Pre, Agg+, Agg-)	0.02	0.984	(2, 101)
EDF 4m	One-way ANOVA	Group (Pre, Agg+, Agg-)	1.24	0.294	(2, 87)
EDF 4n	One-way ANOVA	Group (Pre, Agg+, Agg-)	0.66	0.517	(2, 86)
EDF 4o	One-way ANOVA	Group (Pre, Agg+, Agg-)	0.68	0.512	(2, 83)
EDF 4p	One-way ANOVA	Group (Pre, Agg+, Agg-)	0.26	0.769	(2, 80)
EDF 4q	One-way ANOVA	Group (Pre, Agg+, Agg-)	0.08	0.921	(2, 140)
EDF 4r	One-way ANOVA	Group (Pre, Agg+, Agg-)	1.58	0.21	(2, 117)
EDF 4s	Linear regression	Sag (mV)	2.45	0.026	(1, 16)
EDF 4t	Mixed linear model	Sag (mV), Mouse ID as Group	2.54	0.011	NA

EDF 4v	Fisher's exact test	Group (Gal+, Gal-)	0.63	1	NA
EDF 4w	U test	Group (Gal+, Gal-)	44	0.962	NA
EDF 4x	U test	Group (Gal+, Gal-)	65	0.111	NA
EDF 4y	U test	Group (Gal+, Gal-)	34	0.615	NA
EDF 4z	U test	Group (Gal+, Gal-)	36	1	NA
EDF 5a	Fisher's exact test with Benjamini-Hochberg	Group (Agg+, Agg-)	3.68	0.175	NA
EDF 5a	Fisher's exact test with Benjamini-Hochberg	Group (Pre, Agg+)	2.21	0.175	NA
EDF 5a	Fisher's exact test with Benjamini-Hochberg	Group (Pre, Agg-)	0.6	0.442	NA
EDF 5a	Fisher's exact test with Benjamini-Hochberg	Group (Pre, ZD)	0.05	0.0011	NA
EDF 5a	Fisher's exact test with Benjamini-Hochberg	Group (Pre, NPY)	0.21	0.0255	NA
EDF 5a	Fisher's exact test with Benjamini-Hochberg	Group (Agg+, KD-Y)	2.29	0.48	NA
EDF 5b	One-way ANOVA	Group (Pre, Agg+, Agg-)	0.37	0.692	(2, 164)
EDF 5b	U test with Benjamini-Hochberg	Group (Pre, ZD)	362	0.151	NA
EDF 5b	U test with Benjamini-Hochberg	Group (Pre, NPY)	732	0.725	NA
EDF 5b	U test with Benjamini-Hochberg	Group (Agg+, KD-Y)	46	0.000221	NA
EDF 5c	One-way ANOVA	Group (Pre, Agg+, Agg-)	3.43	0.0352	(2, 141)
EDF 5c	Tukey post hoc test	Group (Agg+, Agg-)	-160.93	0.0669	141
EDF 5c	Tukey post hoc test	Group (Agg+, Pre-)	-143.06	0.0354	141
EDF 5c	Tukey post hoc test	Group (Agg-, Pre)	17.87	0.941	141
EDF 5c	U test with Benjamini-Hochberg	Group (Pre, ZD)	165	0.00285	NA
EDF 5c	U test with Benjamini-Hochberg	Group (Pre, NPY)	568	0.992	NA
EDF 5c	U test with Benjamini-Hochberg	Group (Agg+, KD-Y)	145	0.307	NA
EDF 5e	Chi-square test	Group (Agg+, Ant-Y, Ant-Y1, Ant-Y2)	3.87	0.276	3
EDF 5f	One-way ANOVA	Group (Agg+, Ant-Y, Ant-Y1, Ant-Y2)	1.55	0.211	(3, 62)
EDF 5g	One-way ANOVA	Group (Agg+, Ant-Y, Ant-Y1, Ant-Y2)	1.84	0.151	(3, 59)
EDF 5h	One-way ANOVA	Group (Agg+, Ant-Y, Ant-Y1, Ant-Y2)	0.97	0.412	(3, 58)
EDF 5k	U test	Group (Pre, Post)	1633.5	0.0447	NA
EDF 5l	U test	Group (Pre, Post)	1069	0.162	NA
EDF 5m	U test	Group (Pre, Post)	2069	0.0416	NA
EDF 5n	U test	Group (Pre, Post)	2069	0.0416	NA
EDF 5o	Fisher's exact test	Group (Pre, Post)	0.58	0.255	NA
EDF 5r	U test	Group (Pre, Post)	261.5	0.0145	NA
EDF 5s	U test	Group (Pre, Post)	93	0.0361	NA
EDF 5t	U test	Group (Pre, Post)	297	0.189	NA
EDF 5u	U test	Group (Pre, Post)	297	0.189	NA
EDF 5v	Fisher's exact test	Group (Pre, Post)	0.66	0.518	NA
EDF 5y	One-way ANOVA	Group (P, E, M, D)	0.23	0.875	(3, 121)
EDF 5z	Chi-square test	Group (P, E, M, D)	2.45	0.874	3

EDF 5aa	One-way ANOVA	Group (P, E, M, D)	1.1	0.354	(3, 99)
EDF 5ab	One-way ANOVA	Group (P, E, M, D)	0.29	0.834	(3, 122)
EDF 5ac	Chi-square test	Group (P, E, M, D)	7.54	0.0566	8
EDF 5ad	Linear regression	Neurons with sag	9.35	0.002	(1 , 226)
EDF 5ae	One-way ANOVA	Group (P, E, M, D)	0.44	0.727	(3, 68)
EDF 5af	One-way ANOVA	Group (P, E, M, D)	1.49	0.224	(3, 98)
EDF 5ag	One-way ANOVA	Group (P, E, M, D)	1.65	0.186	(3, 70)
EDF 5ah	One-way ANOVA	Group (P, E, M, D)	0.54	0.655	(3, 72)
EDF 5ai	One-way ANOVA	Group (P, E, M, D)	1.13	0.345	(3, 87)
EDF 5aj	One-way ANOVA	Group (P, E, M, D)	0.4	0.752	(3, 71)
EDF 6b	U test	Group (Pre, AgRP)	1223.5	0.06	NA
EDF 6c	Fisher's exact test	Group (Pre, AgRP)	0.74	0.559	NA
EDF 6d	U test	Group (Pre, AgRP)	884	0.721	NA
EDF 6e	U test	Group (Pre, AgRP)	631	0.255	NA
EDF 6f	U test	Group (Pre, AgRP)	1031.5	0.035	NA
EDF 6h	U test	Group (Pre, NPY)	995	0.016	NA
EDF 6i	Fisher's exact test	Group (Pre, NPY)	0.21	0.017	NA
EDF 6j	U test	Group (Pre, NPY)	732	0.725	NA
EDF 6k	U test	Group (Pre, NPY)	568	0.992	NA
EDF 6l	U test	Group (Pre, NPY)	806	0.0201	NA
EDF 6s	Unpaired t-test	Group (KD-A, ctrl)	-5.8	0.00115	6
EDF 6t	U test	Group (Agg+, KD-A)	171	0.0811	NA
EDF 6u	U test	Group (Agg+, KD-A)	252.5	0.336	NA
EDF 6v	Fisher's exact test	Group (Agg+, KD-A)	0.67	0.565	NA
EDF 6x	U test	Group (Agg+, KD-A)	160	0.0468	NA
EDF 6y	U test	Group (Agg+, KD-A)	318	0.729	NA
EDF 6z	log-rank test	Group (KD-A, ctrl)	0.05	0.411	1
EDF 6aa	Linear regression	Number of KD cells	10.86	0.0459	(1, 3)
EDF 6ab	U test	KD-Y Group (Agg+, Agg-)	7.5	1	NA
EDF 6ab	U test	KD-A Group (Agg+, Agg-)	3.5	1	NA
EDF 6ab	U test	ctrl Group (Agg+, Agg-)	4	0.0755	NA
EDF 6ac	U test	Group (ZD, FD)	28.5	0.0373	NA
EDF 6ad	log-rank test	Group (ZD, FD)	4.96	0.026	1
EDF 6ae	U test	Group (ZD, ctrl)	3	0.222	NA
EDF 7d	One-way ANOVA	Group (P, E, M, D)	0.68	0.584	(3, 12)
EDF 7e	One-way ANOVA	Group (P, E, M, D)	3.04	0.0793	(3, 12)
EDF 8c	Paired t-test	Group (Pre, Post)	0.72	0.505	5
EDF 9a	Paired t-test	Group (Pre+, Agg+)	3.89	0.0302	3
EDF 9b	Paired t-test	Group (Pre-, Agg-)	-1	0.5	1

EDF 9c	Wilcoxon signed-Rank test	Group (Pre+, Agg+)	5715	0.649	NA
EDF 9d	Wilcoxon signed-Rank test	Group (Pre+, Agg+)	2347	0.655	NA
EDF 9e	U test	Group (Agg+, Agg-), window = 4s	9073	0.0022	NA
EDF 9f	Two-way ANOVA	Group (Agg+, Agg-)	109.87	8.55E-20	1
EDF 9f	Two-way ANOVA	Time	2139.71	5.40E-93	1
EDF 9f	Two-way ANOVA	Interaction	127.79	5.06E-22	1
EDF 9f	Two-way ANOVA	Residual	–	–	156
EDF 9g	U test	Group (Agg+, Agg-), window = 2s	5395	0.00841	NA
EDF 9h	Two-way ANOVA	Group (Agg+, Agg-)	0.76	0.385	1
EDF 9h	Two-way ANOVA	Time	1097.77	6.32E-47	1
EDF 9h	Two-way ANOVA	Interaction	4.72	0.0329	1
EDF 9h	Two-way ANOVA	Residual	–	–	76
EDF 9i	U test	Group (Agg+, Agg-), window = 5s	4455	0.00235	NA
EDF 9j	Two-way ANOVA	Group (Agg+, Agg-)	39.42	2.16E-09	1
EDF 9j	Two-way ANOVA	Time	1388.62	6.79E-91	1
EDF 9j	Two-way ANOVA	Interaction	34.27	1.99E-08	1
EDF 9j	Two-way ANOVA	Residual	–	–	196
EDF 9k	U test	Group (Agg+, Agg-), window = 3s	5592	4.40E-11	NA
EDF 9l	Two-way ANOVA	Group (Agg+, Agg-)	0.38	0.54	1
EDF 9l	Two-way ANOVA	Time	947.43	1.21E-57	1
EDF 9l	Two-way ANOVA	Interaction	1.91	0.17	1
EDF 9l	Two-way ANOVA	Residual	–	–	116
EDF 9m	Mixed linear model with Benjamini-Hochberg	Group (Pre+, Agg+), Mouse ID as Group	-0.03	0.976	NA
EDF 9m	Mixed linear model with Benjamini-Hochberg	Group (Pre-, Agg-), Mouse ID as Group	3.75	0.000555	NA
EDF 9m	Mixed linear model with Benjamini-Hochberg	Group (Agg+, Agg-), Mouse ID as Group	3.64	0.000555	NA
EDF 9m	Mixed linear model with Benjamini-Hochberg	Group (Pre+, Pre-), Mouse ID as Group	-0.35	0.969	NA
EDF 9n	Mixed linear model with Benjamini-Hochberg	Group (Pre+, Agg+), Mouse ID as Group	-0.27	0.785	NA
EDF 9n	Mixed linear model with Benjamini-Hochberg	Group (Pre-, Agg-), Mouse ID as Group	5.7	4.86E-08	NA
EDF 9n	Mixed linear model with Benjamini-Hochberg	Group (Agg+, Agg-), Mouse ID as Group	1.64	0.136	NA
EDF 9n	Mixed linear model with Benjamini-Hochberg	Group (Pre+, Pre-), Mouse ID as Group	-1.72	0.136	NA
EDF 9o	Mixed linear model with Benjamini-Hochberg	Group (Pre+, Agg+), Mouse ID as Group	1.83	0.135	NA
EDF 9o	Mixed linear model with Benjamini-Hochberg	Group (Pre-, Agg-), Mouse ID as Group	1.98	0.135	NA

EDF 9o	Mixed linear model with Benjamini-Hochberg	Group (Agg+-, Agg-), Mouse ID as Group	0.48	0.843	NA
EDF 9o	Mixed linear model with Benjamini-Hochberg	Group (Pre+-, Pre-), Mouse ID as Group	-0.04	0.968	NA
EDF 9p	One-way ANOVA	Group (pup vs pup; pup vs food; food vs food)	101.71	2.69E-13	(2, 27)
EDF 9p	Tukey post hoc test	Group (pup vs pup; pup vs food)	4.16	7.40E-11	27
EDF 9p	Tukey post hoc test	Group (pup vs pup; food vs food)	-0.16	0.939	27
EDF 9p	Tukey post hoc test	Group (pup vs food; food vs food)	-4.33	3.13E-11	27
EDF 9r	Kurtosis test	Sniff	-6.32	2.70E-10	NA
EDF 9r	Kurtosis test	Groom	-9.52	1.80E-21	NA
EDF 9r	Kurtosis test	Attack	1.48	0.138	NA
EDF 9s	Paired t-test	Group (Behaviour, state)	-4.03	7.64E-05	242
EDF 9u	Linear regression	PC2 loading	135	4.46E-25	(1, 241)
EDF 9w	Paired t-test	Group (State, Behav)	4.96	0.0077	4
EDF 9x	One-way ANOVA	Group (Sniff, Groom, Attack)	17.2	0.000299	(2, 12)
EDF 9x	Tukey post hoc test	Group (Attack, Sniff)	-0.55	0.0002	12
EDF 9x	Tukey post hoc test	Group (Attack, Groom)	-0.34	0.0099	12
EDF 9x	Tukey post hoc test	Group (Sniff, Attack)	0.21	0.104	12
EDF 9y	One-way ANOVA	Group (0, 1, 2, 3 attack episodes)	8.73	0.007	(4, 7)
EDF 9z	Linear regression	Mean baseline	9.67	0.011	(1, 10)
EDF 9aa	Repeated-measures ANOVA with Greenhouse-Geisser	Group (0, 1, 2, 3 attack episodes)	13.42	0.0352	(3, 9)
EDF 10a	Mixed linear model	Pre pup sniff tuning, Mouse ID as Group	-1.27	0.204	NA
EDF 10b	Mixed linear model	Pre pup groom tuning, Mouse ID as Group	6.49	8.37E-11	NA
EDF 10c	Mixed linear model	Post pup sniff tuning, Mouse ID as Group	1.77	0.077	NA
EDF 10d	Mixed linear model	Post pup groom tuning, Mouse ID as Group	9.17	4.84E-20	NA
EDF 10k	U test	Group (Female, Male)	12	0.172	NA

Supplementary Note

Li et al. (2019) reported direct GABAergic connections from Arc^{AgRP} neurons onto 30% of MPOA neurons in female mice, using channelrhodopsin-assisted circuit mapping (CRACM)[86]. However, as the functional connectivity experiments in Fig. 2C of Li et al. (2019) were not performed with the sodium channel blocker tetrodotoxin (TTX) in the bath solution, it remains unclear whether the presented responses are monosynaptic. Indeed, the reported latencies of inhibitory postsynaptic currents (IPSCs) in females (mean ~8 ms, Fig. 2D) do not, by themselves, support direct connectivity. In addition, the current traces in Fig. 2C of Li et al. (2019) indicate that the observed IPSCs are OFF-responses, i.e., occurring time-locked with the offset of optogenetic stimulation. Using CRACM with the same stimulation parameters as in Li et al. (2019), we were unable to detect direct GABAergic Arc^{AgRP}→MPOA connections even though we could optogenetically evoke action potentials in ChR2-expressing Arc^{AgRP} cells in the same brain slices (Extended Data Fig. 6m–p). While we can only speculate about the reason(s) underlying this discrepancy, our findings do not support the existence of extensive monosynaptic GABAergic Arc^{AgRP}→MPOA connectivity.

References

82. Bergman, M. D. *et al.* Up-regulation of the uterine estrogen receptor and its messenger ribonucleic acid during the mouse estrous cycle: the role of estradiol. *Endocrinology* **130**, 1923–1930 (1992).
83. Michael, S. D. Plasma prolactin and progesterone during the estrous cycle in the mouse. *Proc. Soc. Exp. Biol. Med. Soc. Exp. Biol. Med. N. Y. N* **153**, 254–257 (1976).
84. Murr, S. M., Bradford, G. E. & Geschwind, I. I. Plasma Luteinizing Hormone, Follicle-Stimulating Hormone and Prolactin During Pregnancy in the Mouse1. *Endocrinology* **94**, 112–116 (1974).
85. Barkley, M. S., Geschwind, I. I. & Bradford, G. E. The Gestational Pattern of Estradiol, Testosterone and Progesterone Secretion in Selected Strains of Mice. *Biol. Reprod.* **20**, 733–738 (1979).
86. Li, X.-Y. *et al.* AGRP Neurons Project to the Medial Preoptic Area and Modulate Maternal Nest-Building. *J. Neurosci.* **39**, 456–471 (2019).
