# Supplementary material for: Integration of hunger and hormonal state gates infant-directed aggression
Source: Nature. 2025 Oct 22;648(8092):138–45. doi: 10.1038/s41586-025-09651-2 (PMC12675289; doi:10.1038/s41586-025-09651-2)
Supplement: Supplementary file 2 — Reporting Summary [file 41586_2025_9651_MOESM2_ESM.pdf]

nature portfolio

Corresponding author(s): Johannes Kohl

Last updated by author(s): Sep 1, 2025

Reporting Summary

Nature Portfolio wishes to improve the reproducibility of the work that we publish. This form provides structure for consistency and transparency in reporting. For further information on Nature Portfolio policies, see our Editorial Policies and the Editorial Policy Checklist.

Statistics

For all statistical analyses, confirm that the following items are present in the figure legend, table legend, main text, or Methods section.

n/a	Confirmed	
☐	☒	The exact sample size (*n*) for each experimental group/condition, given as a discrete number and unit of measurement
☐	☒	A statement on whether measurements were taken from distinct samples or whether the same sample was measured repeatedly
☐	☒	The statistical test(s) used AND whether they are one- or two-sided<br>*Only common tests should be described solely by name; describe more complex techniques in the Methods section.*
☐	☒	A description of all covariates tested
☐	☒	A description of any assumptions or corrections, such as tests of normality and adjustment for multiple comparisons
☐	☒	A full description of the statistical parameters including central tendency (e.g. means) or other basic estimates (e.g. regression coefficient) AND variation (e.g. standard deviation) or associated estimates of uncertainty (e.g. confidence intervals)
☐	☒	For null hypothesis testing, the test statistic (e.g. *F*, *t*, *r*) with confidence intervals, effect sizes, degrees of freedom and *P* value noted<br>*Give P values as exact values whenever suitable.*
☒	☐	For Bayesian analysis, information on the choice of priors and Markov chain Monte Carlo settings
☒	☐	For hierarchical and complex designs, identification of the appropriate level for tests and full reporting of outcomes
☐	☒	Estimates of effect sizes (e.g. Cohen's *d*, Pearson's *r*), indicating how they were calculated

Our web collection on statistics for biologists contains articles on many of the points above.

Software and code

Policy information about availability of computer code

Data collection	Ethovision XT 14 software (Noldus) was used for animal tracking. Videos were acquired using custom routines in Bonsai 2.9.0 (NeuroGEARS, https://bonsai-rx.org/) and behaviours were scored using BORIS v. 9.6.4 (https://www.boris.unito.it/). Widefield Images were acquired on a Vectra Polaris Automated Quantitative Pathology Imaging System (Akoya Biosciences) using Phenochart and inForm software (Akoya) for ROI selection and spectral unmixing. Stitching of spectrally unmixed image tiles was performed in QuPath-0.5.1. Confocal images were acquired on a Zeiss LSM 710 via ZEN 2.3 software. Slice electrophysiology data were acquired using pCLAMP 10.6.2 (Scientifica). Fibre photometry was performed on a P3001 fibre photometry system (Neurophotometrics) via Bonsai. Miniature microscopy imaging data were acquired using nVista HD 2.0 software (Inscopix).
Data analysis	Analysis of widefield images was performed in QuPath-0.5.1. Analysis of confocal images was performed in ImageJ (2.16.0). The ImageJ plugin ABBA 0.10.4 (https://abba-documentation.readthedocs.io/en/latest/) was used to register coronal brain sections to the Allen Brain Atlas. Mass spec data were analysed using MANIC software version 3.0.20. Analysis of slice electrophysiology data was performed with Clampfit 10 software (Molecular Devices), WinEDR v4, WinWCP v5 (http://spider.science.strath.ac.uk/sipbs/software_ses.htm), and custom routines written in Python 3.7. Analysis of fibre photometry data was performed in Python 3.7. Preprocessing of miniature microscopy data was performed in Inscopix Data Processing Software v1.5.1 (Inscopix) and further analysis performed in Python 3.7. Statistical analyses were performed in Python 3.7. Code created for this study is available at GitHub (https://github.com/FrancisCrickInstitute/negative_parental_switch).

For manuscripts utilizing custom algorithms or software that are central to the research but not yet described in published literature, software must be made available to editors and reviewers. We strongly encourage code deposition in a community repository (e.g. GitHub). See the Nature Portfolio guidelines for submitting code & software for further information.

Data

Policy information about availability of data

All manuscripts must include a data availability statement. This statement should provide the following information, where applicable:

- Accession codes, unique identifiers, or web links for publicly available datasets
- A description of any restrictions on data availability
- For clinical datasets or third party data, please ensure that the statement adheres to our policy

The data that support the findings of this study are available from the corresponding author upon request. The previously published adult hypothalamus scRNA-seq dataset compiled by Yao et al. (2023) (WMB-10Xv3-HY-log2.h5ad) was downloaded from https://allen-brain-cell-atlas.s3.us-west-2.amazonaws.com/index.html#expression_matrices/WMB-10Xv3/20230630/. Source data are provided with this paper.

Research involving human participants, their data, or biological material

Policy information about studies with human participants or human data. See also policy information about sex, gender (identity/presentation), and sexual orientation and race, ethnicity and racism.

Reporting on sex and gender	No human participants were used in this study
Reporting on race, ethnicity, or other socially relevant groupings	No human participants were used in this study
Population characteristics	No human participants were used in this study
Recruitment	No human participants were used in this study
Ethics oversight	No human participants were used in this study

Note that full information on the approval of the study protocol must also be provided in the manuscript.

Field-specific reporting

Please select the one below that is the best fit for your research. If you are not sure, read the appropriate sections before making your selection.

☒ Life sciences ☐ Behavioural & social sciences ☐ Ecological, evolutionary & environmental sciences

For a reference copy of the document with all sections, see nature.com/documents/nr-reporting-summary-flat.pdf

Life sciences study design

All studies must disclose on these points even when the disclosure is negative.

Sample size	No statistical methods were used to predetermine sample size. Sample sizes were estimated based on previous experiments performed in our group (Kohl et al., 2018, PMID: 29643503; Ammari et al., 2023, PMID: 37797007), and are consistent with those generally used in the field.
Data exclusions	Animals were only excluded if viral transduction was unsuccessful or off-target, or if fibre/cannula/lens tip placement was off-target. For electrophysiological recordings, only cells with a stable series resistance of <30MOhm were analysed.
Replication	The following experiments were replicated twice by different experimenters: switch to pup-directed aggression induced by 6h of food deprivation, slice physiology recordings across estrous cycle. All attempts at replication were successful.
Randomization	Animals were randomly assigned to treatment and control groups. Experimental groups consisted of multiple cohorts to avoid litter and cage effects.
Blinding	Data acquisition was not performed blind. Behavioural data was scored by an individual blind to the experimental design, and analysis of behavioural, histological, electrophysiological and in vivo imaging data was conducted under blind conditions.

Reporting for specific materials, systems and methods

We require information from authors about some types of materials, experimental systems and methods used in many studies. Here, indicate whether each material, system or method listed is relevant to your study. If you are not sure if a list item applies to your research, read the appropriate section before selecting a response.

Materials & experimental systems

n/a	Involved in the study	
☐	☒	Antibodies
☒	☐	Eukaryotic cell lines
☒	☐	Palaeontology and archaeology
☐	☒	Animals and other organisms
☒	☐	Clinical data
☒	☐	Dual use research of concern
☒	☐	Plants

Methods

n/a	Involved in the study	
☒	☐	ChIP-seq
☒	☐	Flow cytometry
☒	☐	MRI-based neuroimaging

Antibodies

Antibodies used

Primary antibodies: rabbit anti c-Fos (Synaptic Systems 226003, 1:2,000), rabbit anti-NPY (Abcam ab30914, 1:500), rabbit anti-AgRP (Abcam ab254558, 1:500); secondary antibodies: donkey anti-rabbit Alexa Fluor-568 (Thermo Fisher A-11057, 1:2,000), donkey anti-rabbit Alexa Fluor-647 (Thermo Fisher A-21245, 1:2,000), goat anti-rabbit Alexa Fluor-647 (Thermo Fisher A-21244, 1:1,000).

Validation

All antibodies used were commercial and validated in previous publications: rabbit anti c-Fos (PMID: 40702175), rabbit anti-NPY (PMID: PMID: 26946128), rabbit anti-AgRP (PMID: 39479445).

Animals and other research organisms

Policy information about studies involving animals; ARRIVE guidelines recommended for reporting animal research, and Sex and Gender in Research

Laboratory animals

C57BL/6J mice from the Crick breeding colonies were used at age 8–14 weeks for all behavioural experiments. Agrp-Cre mice (JAX #012899) were used to target AgRP neurons. For slice physiology experiments, this line was crossed to Rosa26 tdTomato (Ai9, JAX #007909) reporter mice. For hormone receptor KO experiments, Esr1-loxP (estrogen receptor α conditional knockout, imported from EMMA, EM:11179) or PR-loxP (progesterone receptor conditional knockout, see Ammari et al., 2023, PMID: 37797007) were used. All mice were maintained in a C57BL/6J background. Mice had access to food and water ad libitum and were housed on a 12/12 h light-dark cycle (light on: 22:00–10:00) at 21°C and 32% humidity.

House crickets (Grillus domesticus) of either sex and 12–20 mm in length (Northampton Reptile Centre) were used as targets in prey hunting experiments.

Wild animals

No wild animals were used.

Reporting on sex

Experiments were performed in female mice unless indicated in the corresponding figure legends.

Field-collected samples

No field-collected samples were used in this study.

Ethics oversight

All animal procedures performed in this study were approved by the UK government (Home Office) and by the Crick Institutional Animal Welfare Ethical Review Panel (AWERB).

Note that full information on the approval of the study protocol must also be provided in the manuscript.

Plants

Seed stocks

N/A

Novel plant genotypes

N/A

Authentication

N/A
